# Special Features of Bat Microbiota Differ From Those of Terrestrial Mammals

**DOI:** 10.3389/fmicb.2020.01040

**Published:** 2020-06-03

**Authors:** Dong-Lei Sun, Yi-Zhou Gao, Xing-Yi Ge, Zheng-Li Shi, Ning-Yi Zhou

**Affiliations:** ^1^Wuhan Institute of Virology, Chinese Academy of Sciences, Wuhan, China; ^2^State Key Laboratory of Microbial Metabolism, School of Life Sciences and Biotechnology, Shanghai Jiao Tong University, Shanghai, China; ^3^College of Biology, Hunan University, Changsha, China

**Keywords:** bat, bacterial pathogen, gut microbiota, aerobic microbes, sequencing

## Abstract

Bats (order Chiroptera) are one of the most diverse and widely distributed group of mammals with a close relationship to humans. Over the past few decades, a number of studies have been performed on bat viruses; in contrast, bacterial pathogens carried by bats were largely neglected. As more bacterial pathogens are being identified from bats, the need to study their natural microbiota is becoming urgent. In the current study, fecal samples of four bat species from different locations of China were analyzed for their microbiota composition. Together with the results of others, we concluded that bat microbiota is most commonly dominated by Firmicutes and Proteobacteria; the strict anaerobic phylum Bacteroidetes, which is dominant in other terrestrial mammals, especially humans and mice, is relatively rare in bats. This phenomenon was interpreted as a result of a highly specified gastrointestinal tract in adaptation to the flying lifestyle of bats. Further comparative study implied that bat microbiota resemble those of the order Carnivora. To discover potential bacterial pathogens, a database was generated containing the 16S rRNA gene sequences of known bacterial pathogens. Potential bacterial pathogens belonging to 12 genera were detected such as *Salmonella*, *Shigella*, and *Yersinia*, among which some have been previously reported in bats. This study demonstrated high resolution and repeatability in detecting organisms of rare existence, and the results could be used as guidance for future bacterial pathogen isolation.

## Introduction

Bats (common term for order Chiroptera) are one of the most diverse and widely distributed groups of mammals which are capable of sustained flight and have gained important scientific interest in recent decades. Studies on bat-associated viral pathogens regarded bats as natural reservoirs for many emerging viruses (Calisher et al., 2006; Ge et al., 2012), including some of potential threat to human health (Halpin et al., 2000; Leroy et al., 2005; Li et al., 2005). Besides viruses, pathogenic parasites were also found in bats (Murakoshi et al., 2018). In contrast to previous reports on bat viral pathogens (Zhou et al., 2018), studies on bat-associated bacterial pathogens and their natural commensal microbiota are less common (Muhldorfer et al., 2010; Muhldorfer, 2013). Although limited, several potential pathogenic bacterial strains have been isolated or identified in bats over the past few decades, emphasizing the importance of bat bacteria investigations (Muhldorfer, 2013). These potential pathogens include those from genera *Salmonella* (Adesiyun et al., 2009;Reyes et al., 2011), *Shigella* (Arata et al., 1968), *Yersinia* (Muhldorfer et al., 2010), *Vibrio* (Muhldorfer et al., 2011), *Clostridium* (Hajkova and Pikula, 2007), *Campylobacter* (Hazeleger et al., 2010), *Listeria* (Hohne et al., 1975), *Leptospira* (Matthias et al., 2005), and *Pasteurella* (Helmick et al., 2004). Among these species, some can cause diseases to their hosts or other animals while the others did not show obvious pathogenicity and may be considered as opportunistic pathogens (Muhldorfer, 2013).

Besides bacterial pathogens, studies on the natural microbial composition of bats are also scant. A review on bacteria of bats emphasized this point and called for more research efforts on this area (Muhldorfer, 2013). Earlier microbial ecological studies of bat microbiota using culture-based methods have been performed decades ago (Klite, 1965; Heard et al., 1997; Di Bella et al., 2003). The microbial composition of bats may be influenced by their feeding strategies (Carrillo-Araujo et al., 2015; Li et al., 2018) or their ages (Vengust et al., 2018). However, previous studies did not focus on the differences in gut microbiome between bats and land mammals. Moreover, it is now well known that a large number of microbes are unculturable under laboratory conditions, and culture-independent methods have been developed intensively in the last two decades (Muyzer et al., 1993; Ley et al., 2008). Using cloning and sequencing technology, Ley et al. (2008) studied the gut microbiota of 59 mammalian species including two Chiroptera species (*Carollia perspicillata* and *Pteropus giganteus*). Due to the low throughput of this technology, they were only able to acquire 274 and 228 sequences from each bat species. Later, a more comprehensive sequencing-based study of bat microbiota was performed by Phillips et al. (2012) on bats from North America. However, they did not compare bat microbiota with other mammals or focus on bacterial pathogens.

In the current study, gut microbiota of four bat species belonging to four different families were investigated. The four species of bats come from different habitats of China, including a cave in mountains in Yunnan (*Eonycteris spelaea*), a cave in mountains located at Xianning city periphery in Hubei (*Hipposideros armiger*), a cave in a mountain in Guangxi (*Taphozous melanopogon*), and a historic building in the city of Tianjin (*Myotis petax*). Bats of *E. spelaea* are herbivores feeding on banana, longan, etc., while the other three species are insectivorous. This study describes the general features of bat natural microbiota and compared with microbiota of other terrestrial mammals. Moreover, a database containing 16S rRNA gene sequences of known bacterial pathogens was generated to predict potential bacterial pathogens.

## Materials and Methods

### Sample Collection and Phylogenetic Determination

**FIGURE 1:**
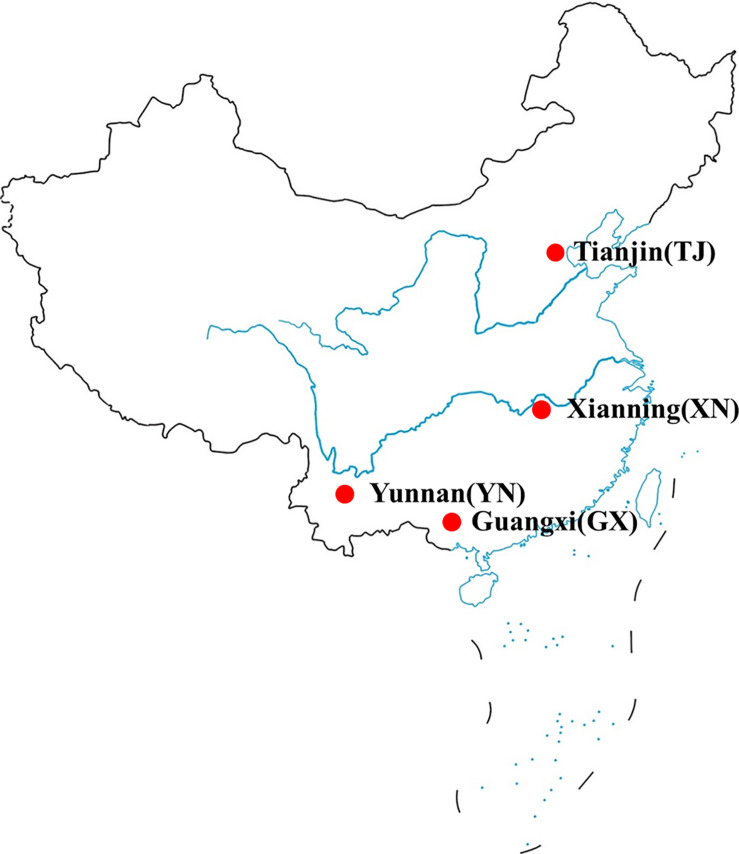
A map showing the four bat sample collection locations in China.

Fecal samples of four wild bat species located at different habitats of China were collected in summer without disturbing their natural life (Figure 1). All animals included this study displayed no apparent clinical signs of illness based on their normal behavior as well as smooth fur appearance. The sampling process, in brief, was performed by spreading an aseptic plastic cloth under bat colonies and waiting until sufficient fecal samples to be collected (normally 1–2 h) (Fofanov and Furstenau, 2018). Samples were frozen in liquid nitrogen immediately after collection. Feces from the same bat species were eventually mixed for two reasons. (1) Sampling of the gregarious animal individually without capturing is difficult. (2) Variations within same species can be minimized by mixing, and repeatability of techniques can be measured. Before this experiment, long-term viral monitor research for these bat colonies had been done, and few bats for each colony were captured and morphologically identified. Two hundred pellets of the same bat species were mixed in the lab.

The phylogenetic relationship was determined by sequencing of cytochrome b (*Cyto b*) gene (please see below). A phylogenetic dendrogram demonstrating the relationship of bat species was constructed using MEGA 5.2 (Tamura et al., 2013). The alignment data sets were used to generate the phylogenetic trees under the maximum likelihood method with a bootstrap value of 1,000 replicates.

### DNA Extraction

Genomic DNA from fecal samples was extracted using a QIAamp DNA stool mini kit (Qiagen, Germany) following the manufacturer’s instructions; a bead-beating step was introduced to facilitate bacteria lysis (Yu and Morrison, 2004). Concentration and purity of extracted DNA were tested on NanoDrop (Thermo, United States). For each species, three aliquots of samples (0.2 g each) were processed simultaneously.

### DNA Sequencing and Data Analysis

Briefly, a 3-mm wing membrane biopsy specimen from wild bats was obtained for DNA extraction by the DNeasy Tissue Kit (QIAGEN). The primers L14724ag-F (5′-ATGATATGAAAAACCATCGTTG-3′) and H15915ag-R (5′-TTTCCNTTTCTGGTTTACAAGAC-3′) were used for amplification of the mitochondrial *Cyto b* gene of bat (Guillén-Servent and Francis, 2006). The PCR amplification was performed as follows: 94°C for 4 min followed by 40 cycles consisting of 94°C for 30 s, 60°C for 30 s, 68°C for 1.5 min, and a final extension of 68°C for 10 min. Degenerate primers F515 (5′-GTGNCAGCMGCCGCGGTAA-3′) and R926 (5′-CCGYCAATTYMTTTRAGTTT-3′) (Quince et al., 2011) targeting the V4–V5 hypervariable region of 16S rRNA genes was used as this region has been shown to have the least intragenomic heterogeneity (Sun et al., 2013) and thus is less likely to overestimate microbial diversity. A 10-nucleotide barcode attached to primer F515 was designed to distinguish different samples in multiplexed sequencing (Table 1). Every two barcodes contained at least two different nucleotides. A 50 μl PCR mixture contained 2.5 U rTaq DNA polymerase (Takara, China), 0.2 mM dNTPs, 0.2 μM each primer, and 50 ng of total genomic DNA. PCR was performed under the following conditions: initial denaturation at 95°C for 5 min, followed by 29 cycles at 95°C for 30 s, 55°C for 30 s, and 72°C for 30 s, with a final extension at 72°C for 10 min. PCR products were further purified using Cycle Pure Kit (OMEGA, United States). Sequencing was performed on the ROCHE 454 FLX Titanium platform (Roche, Switzerland) at the National Human Genome Center of China at Shanghai, China. Sequencing began from forward primer F515 so as to capture the V4 and most of the V5 region.

**TABLE 1:** Bat species included for pyrosequencing, barcodes and number of reads for each sample.

**Family**	**Species**	**Feeding habit**	**Location**	**Barcode**	**Total reads**
*Emballonuridae*	*Taphozous melanopogon*	Insectivorous	Guangxi, China	GX1: CGTAGACTAG	8,258
				GX2: CGTCTAGTAC	7,802
				GX3: CGTGTCTCTA	8,149
*Vespertilionidae*	*Myotis petax*	Insectivorous	Tianjin, China	TJ1: CTCGCGTGTC	7,528
				TJ2: CACACACACT	6,993
				TJ3: CACACGTGAT	8,260
*Hipposideridae*	*Hipposideros armiger*	Insectivorous	Xianning, China	XN1: CATAGTAGTG	8,349
				XN2: CGACGTGACT	7,547
				XN3: CGAGAGATAC	8,262
*Pteropodidae*	*Eonycteris spelaea*	Frugivorous	Yunnan, China	YN1: ATCAGACACG	4,687
				YN2: CACGCTACGT	8,283
				YN3: CAGTAGACGT	8,596

Sequencing raw data (.sff file) was first denoised using the PyroNoise algorithm (Quince et al., 2009) and assigned to each sample according to the 10-nt barcode. Reads without a valid barcode or shorter than 300 bp were excluded from further study. Chimeras were identified and removed using the UCHIME program embedded in MOTHUR (Schloss et al., 2009). Operational taxonomic units (OTUs) were clustered at 3% and 5% dissimilarity levels using the furthest neighbor method and were further used to generate rarefaction curves and estimate richness and diversity indices. The taxonomic assignment of reads was determined by the RDP classifier (Wang et al., 2007) at a confidence threshold of 70%. To compare microbial communities between samples, both the OTU-based Bray–Curtis index and phylogenetic-based unweighted UniFrac (Lozupone et al., 2006) were used. Dendrograms were visualized either by Figtree or EvolView (Zhang et al., 2012). Principal coordinate analysis (PCoA) was also performed using the resulting UniFrac distance matrix. A comparison of the difference of communities was performed by analysis of molecular variance (AMOVA, *p* < 0.05 was considered as significant). The raw data were also transformed into.fastq files with MOTHUR (Schloss et al., 2009) and submitted into GenBank (accession number PRJNA576557). Those who are interested in this can extract effective information with the corresponding barcodes listed in Table 1 from any.fastq files.

### Bacterial Pathogen Detection

A database containing 16S rRNA genes of well-identified bacterial pathogens was constructed from existing pathogen databases including the National Microbial Pathogen Database Resource (NMPDR^1^) and Virulence Factors Database^2^ (Supplementary Table S1). The genes for 16S rRNA in bacterial pathogens were retrieved from the Ribosomal Database Project database^3^. Local BLAST was performed using our sequencing data as the database and the pathogen database as the query. To ensure maximum reliability, only results with sequence identity higher than 99% were recorded.

## Results

### Phylogenetic Analysis of Bat Species

**FIGURE 2:**
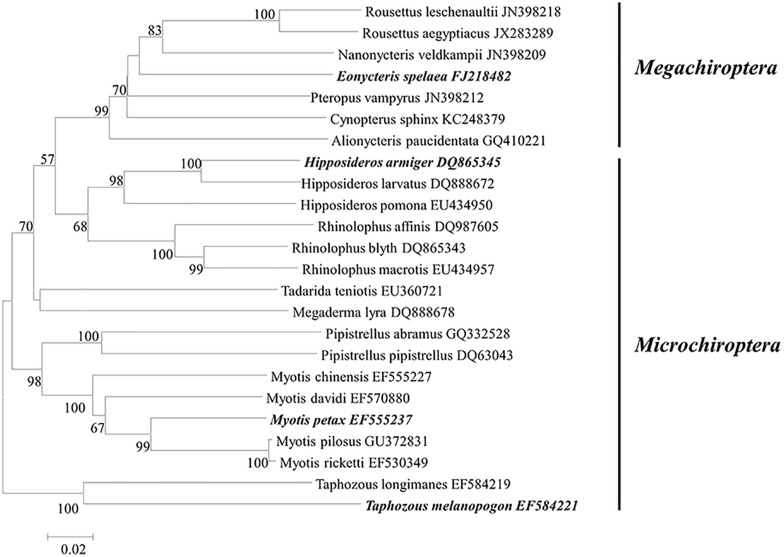
Phylogenetic dendrogram constructed from *Cyto b* gene sequences of bats. According to traditional classification, *Eonycteris spelaea* belong to the Megachiroptera suborder and the others belong to the Microchiroptera suborder.

Phylogenetic information deduced from *Cyto b* gene (Figure 2) showed four bat species each located in a separate cluster. The only frugivorous species *E. spelaea* (YN), although varies significantly in body size and feeding habit, is more closely related to the insectivorous *H. armiger* (XN). *M. petax* (TJ) is more distant from the aforementioned two. The last species *T. melanopogon* (GX) which was located in a distant clade indicated the furthest evolutionary distance from the others.

### Diversity and Richness Estimators Based on Sequencing Data

A total of 126,809 barcoded raw sequences were produced in this study, the ROCHE 454 FLX Titanium platform allowed a mean read length of 421 bp. After strict quality control, trimming of primers and barcodes, and removal of chimeric sequences, 92,714 high-quality sequences with an average of 7,726 reads per sample (range 4,687–8,596, SD = 1,014) proceeded into the downstream process. To minimize its effect on the calculation of diversity and richness estimators, a subsampling procedure was introduced using the smallest sample size and repeated 1,000 times to calculate the average (Schloss et al., 2011). Since a majority of microbial ecological studies have used 3% and 5% dissimilarity levels to define OTUs (McKenna et al., 2008; Nam et al., 2011), this criterion was also used in the current study. At the 3% and 5% dissimilarity levels, the observed OTUs for each species averaged 427 (3% dissimilarity level) (range 346–525, SD = 58) and 197 (5% dissimilarity level) (range 148–256, SD = 36), respectively (Table 2), among which *T. melanopogon* (GX) showed the greatest number of observed OTUs and *M. petax* (TJ) samples showed the least number of observed OTUs at both levels. Chao1 and abundance-based coverage estimator (ACE) estimates of richness were consistent with observed OTUs (Table 2); however, the Shannon diversity index indicated that the frugivorous species of *E. spelaea* of the YN samples (averaged 4.33) was the highest. The result is not surprising because Shannon diversity is a reflection of both richness and evenness. Although YN samples did not show the highest richness, it did have the highest evenness (Table 2). Analysis of rank abundance showed the bat gut microbiota consisted of few highly abundant species and a majority of rare species (Supplementary Figure S1).

**TABLE 2:** Alpha diversity of each species at 3% and 5% dissimilarity levels.

**Sample**	**Observed OTUs**	**Chao1**	**ACE**	**Coverage**	**Shannon index**	**Evenness**
**3% dissimilarity level**						
GX	491 ± 32.8	864 ± 79.3	1,150 ± 126.0	0.948 ± 0.004	4.190 ± 0.304	0.676 ± 0.043
TJ	351 ± 4.4	615 ± 19.9	795 ± 57.7	0.963 ± 0.001	3.682 ± 0.036	0.628 ± 0.005
XN	433 ± 54.6	753 ± 119.6	945 ± 153.0	0.954 ± 0.007	3.443 ± 0.276	0.567 ± 0.034
YN	435 ± 33.8	703 ± 92.4	891 ± 135.8	0.959 ± 0.006	4.330 ± 0.036	0.713 ± 0.013
**5% dissimilarity level**						
GX	247 ± 14.5	417 ± 35.1	542 ± 56.1	0.975 ± 0.003	3.196 ± 0.082	0.580 ± 0.018
TJ	171 ± 8.7	267 ± 9.9	313 ± 38.3	0.985 ± 0.001	3.106 ± 0.076	0.604 ± 0.013
XN	207 ± 17.2	339 ± 36.8	413 ± 80.2	0.981 ± 0.002	2.452 ± 0.255	0.460 ± 0.042
YN	165 ± 26.2	240 ± 54.5	282 ± 93.0	0.987 ± 0.004	3.377 ± 0.046	0.663 ± 0.021

*Standard deviation was calculated from three replicates.*

**FIGURE 3:**
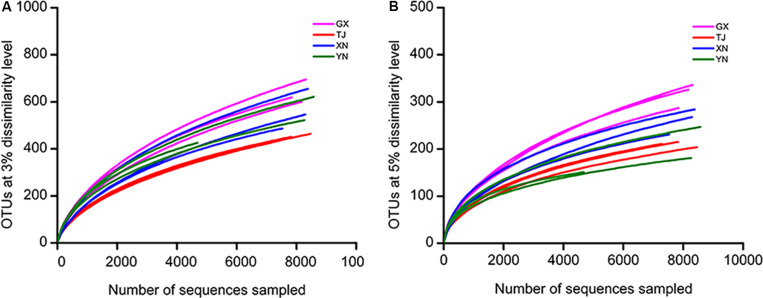
Diversity of bacterial communities as revealed by a rarefaction curve constructed from OTUs at 3% **(A)** and 5% **(B)** dissimilarity levels.

To study whether the sequencing effort is sufficient to recover most of the colony members, a rarefaction analysis was performed based on OTUs clustered at 3% and 5% cutoff (Figures 3A,B). These curves indicated that the sequencing depth of the current study well represented the microbial communities as curves of all samples were approaching plateaus. This conclusion was further consolidated by another parameter reflecting the sequencing completeness good’s coverage which ranged 94–96% among all samples (Table 2). Coverage of this level means no more than six new OTUs were expected for every 100 additional sequencing efforts.

### Taxonomic Assignment of Bacterial Pyrosequencing Reads

**FIGURE 4:**
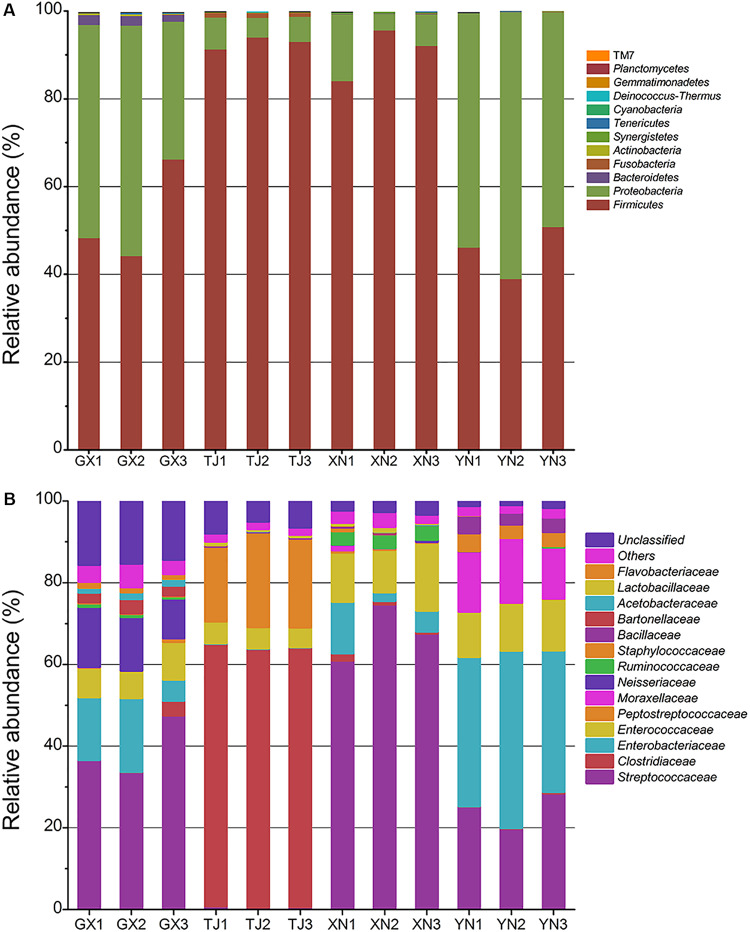
Taxonomic classification of pyrosequencing reads at phylum level **(A)** and family level **(B)** using the RDP classifier at a confidence threshold of 70%.

At a confidence threshold of 70%, 92,525 out of 92,714 (99.7%) high-quality sequences can be assigned to 12 known phyla (Figure 4A). Surprisingly, Firmicutes and Proteobacteria dominated the gut microbiota of all four bat species. Firmicutes, which was also a common composition of human gut flora, took up around one half of the microbial community in GX and YN samples; however, this group was even more dominant in TJ and XN samples with a percentage of more than 90%. Proteobacteria is also commonly discovered in guts of terrestrial mammals (mice, Nava et al., 2011; macaques, McKenna et al., 2008; and humans, Nam et al., 2011), but this phylum is rarely dominant in those mammals (usually less than 5%). In this study, Proteobacteria occupied 44% sequences in GX and 54% in YN and thus can be regarded as dominant. The proportions of this phylum in TJ and XN samples (6% and 9%, respectively) were also higher than those in other terrestrial mammals. To our surprise, another dominant phylum other than Firmicutes in higher mammals, Bacteroidetes, was nearly absent in bats. Only GX samples detected around 2% Bacteroidetes sequences; its proportion in other samples was less than 0.01%. Similarly, TJ samples detected around 1% sequences from Fusobacteria; however, it was hardly detected in the others. Phyla of Actinobacteria, Synergistetes, Tenericutes, Cyanobacteria, Deinococcus–Thermus, Gemmatimonadetes, and TM7 were found to be minority groups.

A more detailed community composition at family level was demonstrated in Figure 4B. GX, XN, and YN samples shared a common feature as they all consisted of a large proportion of sequences from Streptococcaceae, Enterobacteriaceae, and Enterococcaceae. In TJ samples, Clostridiaceae seemed to have taken the place of *Streptococcaceae* and made up more than 60% of total microbiota. Some species-specific groups can be distinguished at this taxonomic level, for example, Neisseriaceae for GX, Peptostreptococcaceae for TJ, Ruminococcaceae for XN, and Moraxellaceae for YN. Further study of this result revealed, at least in some bat species, that their gut flora tends to be dominated by facultative (e.g., Streptococcaceae, Enterobacteriaceae, and Enterococcaceae) or even aerobic (e.g., Neisseriaceae) microorganisms rather than strict anaerobic groups like Bacteroidetes. A heatmap constructed on the major 30 genera (Supplementary Figure S2) showed that *Lactococcus* was consistently detected in all samples, which is reminiscent of a previous report about the slight acidity of bat intestinal contents (Klite, 1965). Another genus detected with considerable proportion among all samples was *Enterococcus*, which is also a common commensal organism of animal guts.

### Community Similarity Analysis

**FIGURE 5:**
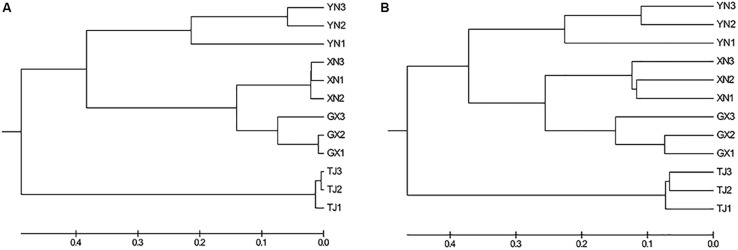
Dendrogram showing the similarity of bacterial communities based on the **(A)** Yue and Clayton measure of dissimilarity and **(B)** Bray–Curtis index.

**FIGURE 6:**
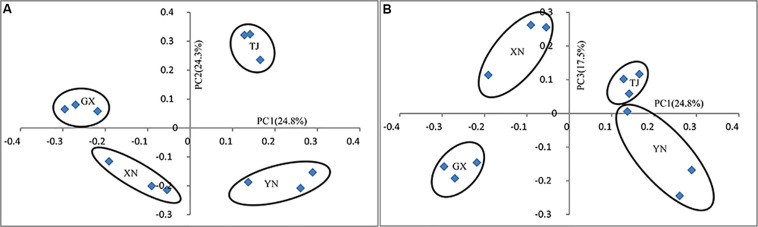
Principal coordinate analysis of UniFrac metric for all samples: **(A)** PC1 and PC2 and **(B)** PC1 and PC3.

Multiple approaches were employed to compare the microbial community similarity of bat species. Dendrogram constructed from the Yue and Clayton measure of dissimilarity and Bray–Curtis index based on OTU information (3% cutoff) both indicated TJ samples were the most divergent from the others; on the other hand, GX and XN samples consistently showed the closest similarity (Figures 5A,B). We also want to question whether the host phylogenetic relationship is in accordance with that of its gut microbiota. However, a comparison between the host evolutionary relationship (Figure 2) and gut flora dendrogram (Figure 5) showed no obvious correlation (congruence test, de Vienne et al., 2007, *p* > 0.05). Since the above OTU-based similarity analysis did not take into consideration the phylogenetic information, unweighted UniFrac was performed, and PCoA was used to study the communities along axes of maximal variance (Figure 6). It is interesting that four species each located in a separate quadrant, and AMOVA concluded that every between-group difference was significant (*p* < 0.05). Three replicates strongly clustered together, indicating good repeatability in this study.

**FIGURE 7:**
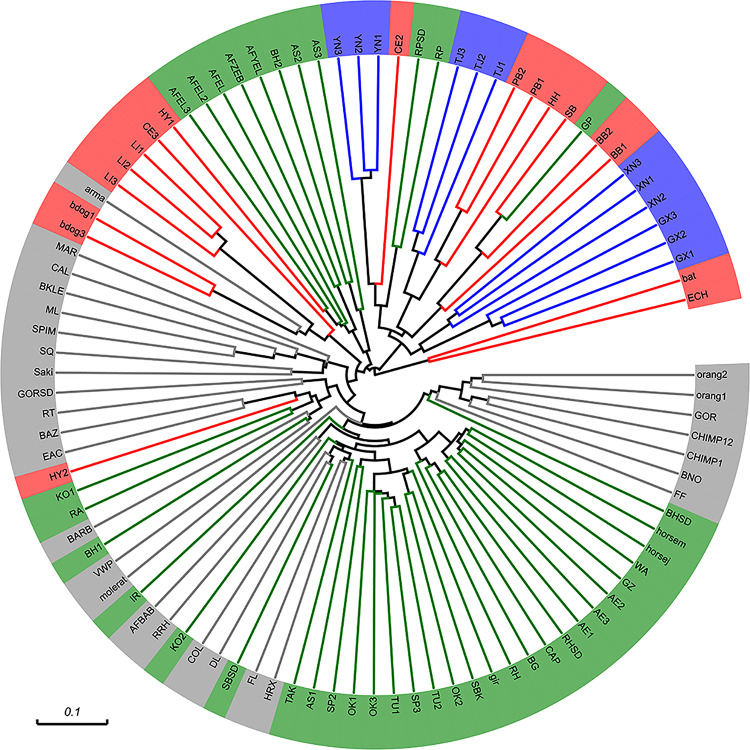
Comparison of bat microbiota with that of other mammals (unweighted UniFrac analysis). Reference sequence data from Ley et al. (2008) were trimmed to match the same position and length of the 16S rRNA gene. Different colors represent different feeding habits (green: herbivorous, red: carnivorous, gray: omnivorous) except blue which marked our sample. Abbreviations of species: orangutans, Orang1,2; western lowland gorillas, GOR and GORSD; chimpanzees, CHIMP1,12; black rhinoceros, BNO; flying fox, FF; bighorn sheep, BHSD, BH1, and BH2; horses, HORSEJ and HORSEM; Somali wild ass, WA; Grevy’s zebra, GZ; Asian elephants, AE1-3; rock hyraxes, RH and RHSD; Capybara, CAP; Bwindi gorilla, BG; giraffe, gir; springboks, SBK; Okapi, OK1-3; Transcaspian Urial sheep, TU1,2; Speke’s gazelles, SP3; Argali sheep, AS1-3; takin, TAK; François langur, FL; springboks, SB and SBSD; douc langur, DL; eastern black and white colobus, COL; red kangaroos, KO1,2; red river hog, RRH; baboons, AFBAB; Indian rhinoceros, IR; naked mole rat, MOLERAT; Visayan warty pig, VWP; babirusa, BARB; spotted hyenas, HY1,2; East Angolan colobus, EAC; ring-tailed lemur, RT; white-faced saki, Saki; Prevost’s squirrel, SQ; spider monkey, SPIM; mongoose lemur, ML; black lemur, BKLE; calimicos (Goeldi’s marmoset), CAL; Geoffrey’s marmoset, MAR; bush dogs, bdog1,3; armadillo, arma; lions, Li1-3; cheetahs, CE2,3; African elephants, AFEL, AFYEL, AFEL2,3; Hartmann’s mountain zebra, AFZEB; red panda, RP and RPSD; polar bears, PB1,2; hedgehog, HH; spectacled bear, SB; giant panda, GP; black bears, BB1,2; Seba’s short-tailed bat, bat; echidna, ECH; rabbit, RA.

To address the question about the position of bat microbiota in the mammalian world, we took advantage of Ley’s data of mammalian gut microbes (Ley et al., 2008). The gut genome of bats is compared with that of terrestrial animals here. Unweighted UniFrac analysis showed bat microbiota was generally restricted to a cluster which contained species almost entirely from order Carnivora (Figure 7). These species include black bear (BB), polar bear (PB), giant panda (GP), red panda (RP and RPSD), and so on. It should be noted that although some species (e.g., giant panda and red panda) are herbivorous, they evolutionary belong to Carnivora and share a similar gut physiology (simple gut compared to foregut or hindgut) with their carnivorous cousins (Ley et al., 2008). The only exception that was not from Carnivora in the cluster was hedgehog (HH) from order *Insectivora*; however, it also has a simple gut type similar to that of the above Carnivora species.

### Potential Bacterial Pathogen Exploration

**FIGURE 8:**
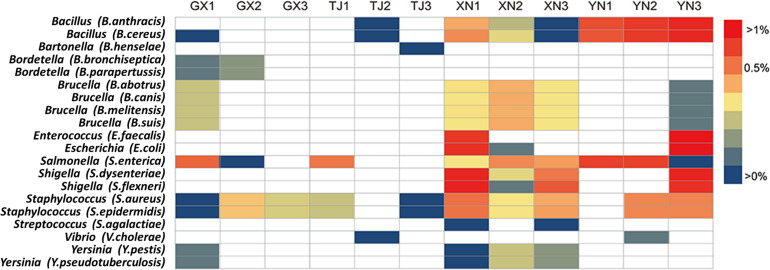
Heatmap of detected potential bacterial pathogens. The strains in brackets represent the pathogenic bacteria used for retrieval and comparison.

Since a considerable number of bacterial pathogens carried by bats have been reported (Muhldorfer, 2013), a 16S rRNA gene database constructed from well-characterized bacterial pathogens was used in an effort to search for potential bacterial pathogens in bats. To maximize reliability, a threshold of 99% sequence identity was applied. Potential pathogens from 12 genera were detected with different proportions (Figure 8), among which *Salmonella*, *Staphylococcus*, and *Bacillus* were consistently detected in all bat species. *Brucella* was detected in three species except TJ. *Shigella*, *Enterococcus*, and *Escherichia* were detected in XN and YN; on the other hand, GX and XN samples showed the existence of *Yersinia*. Other rarely detected but potentially pathogenic genera include *Bartonella*, *Bordetella*, *Streptococcus*, and *Vibrio*.

## Discussion

The order Chiroptera is one of the most diverse and geographically distributed groups of mammals capable of sustained flight (Muhldorfer, 2013). Their wide distribution and close relationship to humans have drawn much research attention over the past few decades (Ciminski et al., 2019; Fan et al., 2019; Laing et al., 2019). A variety of viral pathogens with clinical significance have been identified in bats (Halpin et al., 2000; Leroy et al., 2005; Ge et al., 2012), especially a recently discovered SARS-like coronavirus that might account for the 2003 SARS pandemic in China (Ge et al., 2013). Bats are able to both tolerate viral infections and live well beyond expectations (Hayman, 2019). Compared to the number of studies on bat virus, knowledge about bat bacteria seemed to have lagged behind. Early researches on bat commensal bacteria can be dated back to the 1960s (Klite, 1965); this and later studies of bat microbiota were mostly based on cultivation (Heard et al., 1997). Due to the wide existence of uncultivable microorganisms, those studies were obviously limited. The development of sequencing techniques provided a convenient and powerful approach in studying the composition of extremely complex environments such as the gut (Nam et al., 2011), soil (Roesch et al., 2007), and seawater (Lee et al., 2011).

Traditional opinions classified Chiroptera into Megachiroptera (bigger in size and mostly feed on fruits) and Microchiroptera (echolocating and mostly feeding on insects). From this view, *E. spelaea* belongs to Megachiroptera while the other three belong to Microchiroptera. However, some evidences suggested Chiroptera be classified into Yinpterochiroptera (including *Eonycteris* and *Hipposideros*) and Yangochiroptera suborders (including *Taphozous* and *Myotis*) (Springer et al., 2001; Teeling et al., 2002, 2005). Our phylogenetic evidence based on the *Cyto b* gene sequence supported this theory as *E. spelaea* first joined *H. armiger*; these two together clustered with *M. petax* and *T. melanopogon*.

The current study revealed that strict anaerobic bacteria from Bacteroidetes were almost absent in bats; on the contrary, facultative or even aerobic species were more abundant. This result is surprising because Bacteroidetes has been shown as a dominating group of commensal community in a majority of terrestrial mammals including humans, mice, and many other species (Suchodolski et al., 2008; Nam et al., 2011; Nava et al., 2011; Shanks et al., 2011). Although it has not been pointed out, the gut microbiota of two bat species in Ley’s study were also significantly dominated by Firmicutes or Proteobacteria and lacking Bacteroidetes (Ley et al., 2008). The result is also in consistency with Phillips’ study as Bacteroidetes only made up 1% of the total microbiota (Phillips et al., 2012). We hereby argue this phenomenon is a coevolution of gut flora and host gut physiology because the gut system of bats has many unique features compared to other mammals. The key features to bat gut include, firstly, shorter intestinal length (about one third to one fifth of that of mouse of comparable weight, Klite, 1965; Caviedes-Vidal et al., 2007); secondly, a unique structure of the gastrointestinal system, usually lacking the cecum and appendix (Klite, 1965; Gadelha-Alves et al., 2008) and in some species even the colon (Makanya and Maina, 1994); and thirdly, significantly reduced transit time of food digest through the intestine (as short as 15 min in some species, Klite, 1965, compared to several hours in mice of comparable size). In addition, as the only mammals with a true flying ability, which is the most energy-consuming activity, bats have well adapted to carrying increased oxygen demands of flight and elevated oxygen diffusion capacity (Canals et al., 2005). The metabolic rate of a flying bat is three to five times higher than that of similar-sized terrestrial mammals in maximum exercise (Shen et al., 2010). Further efforts are required to verify whether the lack of a strict anaerobic Bacteroidetes community is universal to other flying animals.

It has been well established that commensal microbiota is affected by a variety of factors including host diet, phylogeny, and gut type (Ley et al., 2008; Zhang et al., 2010). Using culture-dependent methods, Klite (1965) concluded that food habits alone would not seem to account for the structural changes of microbial communities between bat species. A recently published mouse model reported that host diet explained 57% of total variation compared to less than 12% explained by genetic mutation (Zhang et al., 2010). Our result also indicated that the phylogenic relationship did not show clear consistency with microbiota similarity. However, if more species of the same family were included to minimize within-family variation, the influence of a phylogenic relationship might be seen as shown by a previous study (Phillips et al., 2012). Beyond host diet, other geographical and environmental factors may have more significant effects on microbiota. Further investigation may focus on more bat species of diversified feeding habits as well as their living environments to reveal the relationship of microbiota to diet, age, sex, and environmental factors. It should be noted that a bat virus study detected a considerable number of sequences belonging to bacteriophages (Ge et al., 2012); whether bacteriophages have any effect on bat microbiota is also an untouched area.

The studies on bat microbiota have become epidemiologically significant as more potential bacterial pathogens are being identified in bats, including *Salmonella enteritidis* (Muhldorfer et al., 2011), *Yersinia enterocolitica* (Muhldorfer et al., 2010), *Shigella boydii* (Arata et al., 1968), and *Streptococcus dentirousetti* (Takada and Hirasawa, 2008). Some of these species were reported to be able to cause local or systemic diseases in bats or other animals while some others did not show obvious pathogenicity and were regarded as opportunistic pathogens (Muhldorfer, 2013).

Earlier attempts to isolate bacterial pathogens usually require enormous sampling efforts and poor repeatability as pathogens were only isolated in few individuals (Hohne et al., 1975; Hazeleger et al., 2010; Muhldorfer et al., 2011). With higher resolution and better repeatability, the culture-independent technology can be applied to perform preliminary pathogen surveys. Although partial 16S rRNA genes were not able to assign sequences down to species level, the result is still meaningful as a guidance to targeted identification of pathogens. Since each pathogen carries unique DNA or RNA signatures that differentiate it from other organisms, if the database of these signatures can be curated, future metagenomic or transcriptomic studies will provide more detailed and conclusive results about bacterial pathogens (Leon et al., 2018). As an example, the identification of *Bartonella mayotimonensis* from metagenomic research proved the feasibility of this approach (Veikkolainen et al., 2014).

In conclusion, the pyrosequencing analysis of the commensal microbiota of four Chinese bat species revealed key features unique to bat gut flora, including the absence of Bacteroidetes group, which is a dominant group in other terrestrial mammals. Besides that, general bat microbiota is more similar to that of the Carnivora animals. Potential bacterial pathogens from 12 genera were detected, which can be further investigated.

## Data Availability Statement

The datasets generated for this study can be accessed from GenBank, PRJNA576557.

## Ethics Statement

This study was approved by the Animal Ethics Committee of the Wuhan Institute of Virology (Animal Ethics Approval Number: WIVA05201202).

## Author Contributions

D-LS acquired and analysed data, and wrote the manuscript. Y-ZG analysed data and wrote the manuscript. X-YG and Z-LS collected samples and provided technical guidance. N-YZ contributed research ideas and modified the manuscript.

## Conflict of Interest

The authors declare that the research was conducted in the absence of any commercial or financial relationships that could be construed as a potential conflict of interest.

## Footnotes

1 http://www.nmpdr.org

2 http://www.mgc.ac.cn/VFs/main.htm

3 http://rdp.cme.msu.edu/
