# Supplementary material for: Special Features of Bat Microbiota Differ From Those of Terrestrial Mammals
Source: Front Microbiol. 2020 Jun 3;11:1040. doi: 10.3389/fmicb.2020.01040 (PMC7284282; doi:10.3389/fmicb.2020.01040)
Supplement: Supplementary file 1 [file Data_Sheet_1.DOCX]

**Supplement material**

**Special features of bats microbiota differ from terrestrial mammals**

Dong-Lei Sun^1,3#^, Yi-Zhou Gao^2#^, Xing-Yi Ge^1,4#^, Zheng-Li Shi^1^, Ning-Yi Zhou^1,2*^

1. Wuhan Institute of Virology, Chinese Academy of Sciences, Wuhan, China

2. State Key Laboratory of Microbial Metabolism and School of Life Sciences & Biotechnology, Shanghai Jiao Tong University, Shanghai, China

3. Division of Immunology, Virginia-Maryland Regional College of Veterinary Medicine, Maryland Pathogen Research Institute, University of Maryland, College Park, MD, USA.

4. College of Biology, Hunan University, Changsha, China.

(#)These authors contributed equally to this work.

(*)Correspondence to Ning-Yi Zhou ([ningyi.zhou@sjtu.edu.cn](mailto:ningyi.zhou@sjtu.edu.cn))

or Zheng-Li Shi (zlshi@wh.iov.cn)


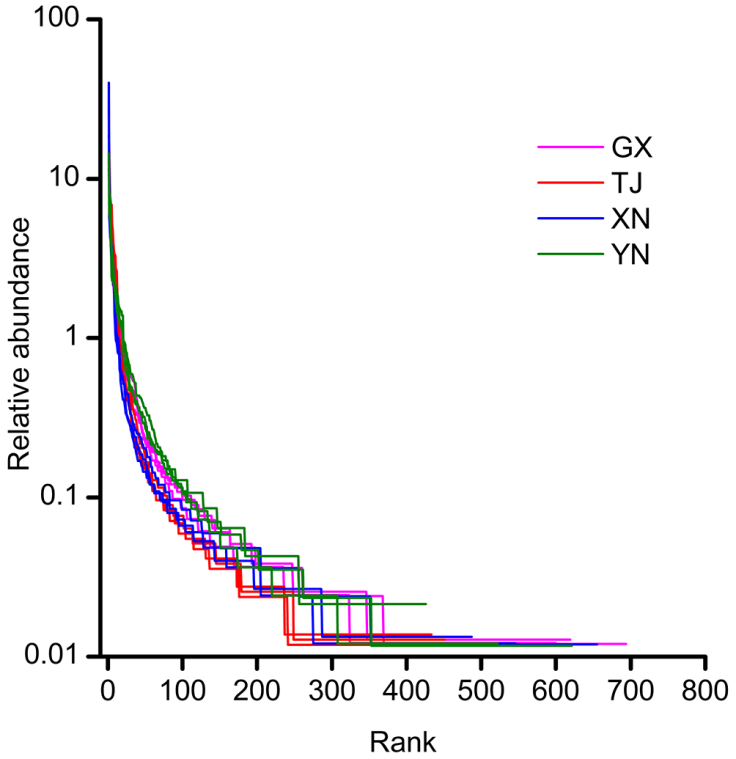


Figure S1. Rank abundance curve based on OTUs at 3% dissimilarity cutoff.


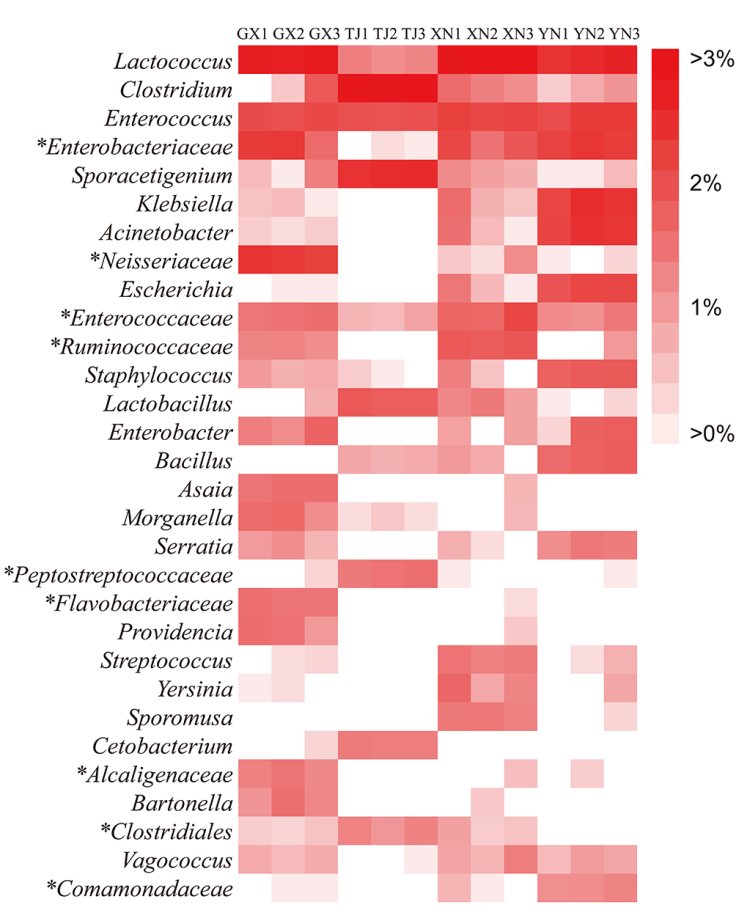


Figure S2. Heatmap of top 30 genera. Asterisk indicates unclassified genera, thus their upper taxonomic level was listed.

Table S1. Identified bacterial pathogens from public databases.

| Species | Pathogenic Disease | Reference |
| --- | --- | --- |
| *Bacillus anthracis* | Etiological Agent For Anthrax | VFDB |
| *Bacillus cereus* | Opportunistic Pathogen Causing Food Poisoning Manifested By Diarrhoeal Or Emetic Syndromes | VFDB |
| *Bartonella bacilliformis* | Carrion's Disease: Oroya Fever And Verruga Peruana | VFDB |
| *Bartonella quintana* | Trench Fever, Endocarditis, Bacillary Angiomatosis | VFDB |
| *Bartonella henselae* | Cat-Scratch Disease | VFDB |
| *Bordetella pertussis* | Etiologic Agent Of Whooping Cough | VFDB |
| *Bordetella parapertussis* | Causes A Milder Form Of Whooping Cough In Human Beings And Chronic, Nonprogressive Pneumonia In Sheep | VFDB |
| *Bordetella bronchiseptica* | Causes Chronic Respiratory Infections In A Wide Range Of Animals | VFDB |
| *Brucella melitensis* | Causative Agents Of Brucellosis | VFDB |
| *Brucella abortus* | Causative Agents Of Brucellosis | VFDB |
| *Brucella suis* | Causative Agents Of Brucellosis | VFDB |
| *Brucella canis* | Causative Agents Of Brucellosis | VFDB |
| *Campylobacter jejuni* | Human Bacterial Gastroenteritis | NMPDR |
| *Campylobacter coli* | Human Bacterial Gastroenteritis | VFDB |
| *Clostridium botulinum* | Botulism | VFDB |
| *Clostridium tetani* | Tetanus | VFDB |
| *Corynebacterium diphtheriae* | Causative Agent Of The Acute, Communicable Disease Diphtheria | VFDB |
| *Corynebacterium jeikeium* | Causative Agent Of A Variety Of Severe Nosocomial Infections | VFDB |
| *Enterococcus faecalis* | Nosocomial Infections | VFDB |
| *Escherichia coli* | Cause Various Diseases In Humans, Including Several Types Of Diarrhea, Urinary Tract Infections, Sepsis, And Meningitis | VFDB |
| *Haemophilus influenzae* | Systemic Infections Such As Bacteraemia, Meningitis, Septic Arthritis And Pneumonia In Young Children | VFDB |
| *Helicobacter pylori* | Gastric And Duodenal Ulcers, The Only Recognized Bacterial Carcinogen | VFDB |
| *Legionella pneumophila* | Legionnaires' Disease | VFDB |
| *Listeria monocytogenes* | Infects Both Human And Aminals Causing Meningitis, Sepsis, And Abortion | NMPDR |
| *Listeria ivanovii* | Causes Septicemic Disease, Neonatal Sepsis In Sheep And Cattle | VFDB |
| *Mycobacterium tuberculosis* | Causative Agents Of Tuberculosis | VFDB |
| *Mycobacterium leprae* | Leprosy | VFDB |
| *Mycobacterium ulcerans* | Buruli Ulcers | VFDB |
| *Neisseria gonorrhoeae* | Gonorrhea | VFDB |
| *Neisseria meningitidis* | Epidemic Meningitis | VFDB |
| *Pseudomonas aeruginosa* | Can Cause A Variety Of Opportunistic Infections | VFDB |
| *Salmonella enterica* | A Leading Cause Of Human Gastroenteritis | VFDB |
| *Shigella dysenteriae* | Shigellosis | VFDB |
| *Shigella flexneri* | Shigellosis | VFDB |
| *Staphylococcus aureus* | Superficial Abscesses And Wound Infections, Osteomyelitis, Endocarditis And Septicaemia | NMPDR |
| *Staphylococcus epidermidis* | Catheter-Associated Infections, Biofilms On Plastic Implants, Endocarditis | VFDB |
| *Streptococcus pyogenes* | Pharyngitis, Scarlet Fever, Impetigo, Erysipelas, etc. | NMPDR |
| *Streptococcus agalactiae* | Neonatal Meningitis, Bacteria Sepsis And Pneumonia | VFDB |
| *Streptococcus pneumoniae* | Pneumonia | NMPDR |
| *Vibrio cholerae* | Human Intestinal Disease Cholera | NMPDR |
| *Yersinia pestis* | The Agent Of Black Death | VFDB |
| *Yersinia pseudotuberculosis* | An Agent Of Mesenteric Adenitis And Septicaemia | VFDB |
| *Yersinia enterocolitica* | Causes Gastrointestinal Syndromes, Ranging From Acute Enteritis To Mesenteric Lymphadenitis | VFDB |
| *Chlamydia trachomatis* | The Most Common Cause Of Bacterial Sexually Transmitted Disease | NMPDR |
| *Mycoplasma mobile* | Usually Cause Chronic Diseases And Persist Intheir Host For Extended Periods After Infection | VFDB |
| *Treponema pallidum* | Syphilis, Congenital syphilis | NMPDR |
| *Rickettsia prowazekii* | Typhus Fever | NMPDR |
| *Borrelia burgdorferi* | Lyme disease | VFDB |
| *Chlamydophila psittaci* | Psittacosis | NMPDR |
| *Francisella tularensis* | Tularemia | NMPDR |
| *Leptospira interrogans* | Leptospirosis | VFDB |

NMPDR: National Microbial Pathogen Database Resource (<http://www.nmpdr.org>)

VFDB: Virulence Factors Database (http://www.mgc.ac.cn/VFs/main.htm)
